# The value of radiomics based on dual-energy CT for differentiating benign from malignant solitary pulmonary nodules

**DOI:** 10.1186/s12880-022-00824-3

**Published:** 2022-05-21

**Authors:** Gao Liang, Wei Yu, Shu-qin Liu, Ming-guo Xie, Min Liu

**Affiliations:** 1 Department of Radiology, Hospital of ChengDu University of Traditional Chinese Medicine, Chengdu, 610075 China; 2 Toxicology Department, WestChina-Frontier PharmaTech Co., Ltd. (WCFP), Chengdu, 610075 China

**Keywords:** Pulmonary nodules, Computed tomography, Dual-energy, Radiomics

## Abstract

**Objective:**

To investigate the value of monochromatic dual-energy CT (DECT) images based on radiomics in differentiating benign from malignant solitary pulmonary nodules.

**Materials and methods:**

This retrospective study was approved by the institutional review board, and informed consent was waived. Pathologically confirmed lung nodules smaller than 3 cm with integrated arterial phase and venous phase (AP and VP) gemstone spectral imaging were retrospectively identified. After extracting the radiomic features of each case, principal component analysis (PCA) was used for feature selection, and after training with the logistic regression method, three classification models (Model_AP_, Model_VP_ and Model_Combination_) were constructed. The performance was assessed by the area under the receiver operating curve (AUC), and the efficacy of the models was validated using an independent cohort.

**Results:**

A total of 153 patients were included and divided into a training cohort (n = 107) and a validation cohort (n = 46). A total of 1130 radiomic features were extracted from each case. The PCA method selected 22, 25 and 35 principal components to construct the three models. The diagnostic accuracy of Model_AP_, Model_VP_ and Model_Combination_ was 0.8043, 0.6739, and 0.7826 in the validation set, with AUCs of 0.8148 (95% CI 0.682–0.948), 0.7485 (95% CI 0.602–0.895), and 0.8772 (95% CI 0.780–0.974), respectively. The DeLong test showed that there were significant differences in the AUCs between Model_AP_ and Model_Combination_ (*P* = 0.0396) and between Model_VP_ and Model_Combination_ (*P* = 0.0465). However, the difference in AUCs between Model_AP_ and Model_VP_ was not significant (*P* = 0.5061). These results demonstrate that Model_Combination_ shows a better performance than the other models. Decision curve analysis proved the clinical utility of this model.

**Conclusions:**

We developed a radiomics model based on monochromatic DECT images to identify solitary pulmonary nodules. This model could serve as an effective tool for discriminating benign from malignant pulmonary nodules in patients. The combination of arterial phase and venous phase imaging could significantly improve the model performance.

## Introduction

The detection rate of pulmonary nodules has increased in recent years with the development of computed tomography (CT) scanning, imaging reconstruction and artificial intelligence technologies [1]. Solitary pulmonary nodules are usually classified as malignant or benign nodules. However, both types of nodules share similar imaging features, such as signs of lobulated and spiculated margins [2, 3]. Thus, it is difficult to differentiate malignant from benign nodules using conventional imaging methods alone. Radiomics can extract a large quantity of imaging features from CT, MRI, PET-CT, or other databases via high-through put extraction and can be used to analyse the correlation between these features and underlying pathological changes of disease [4–6]. Radiomics therefore not only plays an important role in the differentiation of malignant from benign tumours but also in metastasis prediction, pathological grading, malignancy staging, and therapeutic effect monitoring [7–10], as it can reflect the heterogeneity within tumours and improve the diagnostic accuracy.

Gemstone spectral imaging (GSI) allows for the simultaneous acquisition of data at two different energy levels; the concept of GSI is based on the fact that tissues of different compositions exhibit variable attenuation characteristics under different X-ray energy irradiation [11]. Compared to conventional CT, with GSI, the original single-parameter image is transformed into a multiparameter image, and the mixed-energy image is transformed into a single-energy image [12]. GSI is thus one of the most promising imaging modes in clinical practice [13], as it can realize accurate material decomposition images, such as water- and iodine-based material decomposition [14].

In this study, the differences in GSI features between malignant and benign pulmonary nodules were analysed based on spectral CT. Additionally, prediction models were constructed and trained with the logistic regression method to differentiate malignant from benign pulmonary nodules, and the performance of the classifier was quantitatively assessed.

## Materials and methods

### Patients

The review board at our institution (Hospital of Chengdu University of Traditional Chinese Medicine Research Ethics Committee) approved this retrospective study and waived informed consent. All methods were performed in accordance with the relevant guidelines and regulations.

We collected images of patients who had a solitary pulmonary nodule and underwent dual-energy CT scans of the thorax from September 2013 to April 2014. Two radiologists re-evaluated the quality of the GSI and assessed the lesions for inclusion. The criteria for inclusion were as follows: (1) The pulmonary nodule was a single, solid (without pure ground-glass pulmonary nodules), well-circumscribed lesion no more than 30 mm in diameter, which was surrounded by pulmonary parenchyma without atelectasis, pleural nodules or mediastinal lymphadenopathy. (2) The patients underwent dual-period and dual-energy CT examination. (3) The pulmonary nodules were surgically removed or biopsied, and an accurate pathological diagnosis was obtained. The exclusion criteria were as follows: (2) the GSI quality was greatly disturbed by artefacts; and (2) pure ground-glass pulmonary nodules were detected. Eligible patients were randomly assigned to the training cohort and validation cohort at a ratio of 7:3.

### Image acquisition and radiomic feature extraction

Image acquisition and radiomic feature extraction are the basis for subsequent data analysis. Dual-energy CT scans were obtained using a Discovery CT 750HD scanner (GE, Healthcare, 64-detector-row). Dual-energy CT images were acquired at 96 mA for a 120-kVp tube and at 408 mA for an 80-kVp tube and then reconstructed using enhanced monochromatic imaging (70 keV, slice thickness/interval 1.25 mm). An 80–100 ml (2.00 ml/kg of body weight) nonionic iodinated contrast material (Iopamidol, 300 mg/ml, Shanghai Bracco Sine Pharmaceutical Co. Ltd, China) was injected into the vein at a rate of 4.0 ml/s. Dual-phasic (arterial and venous phase) images were obtained at 30 s and 60 s after contrast injection.

Then, the segmentation and extraction of radiomic features from each pulmonary nodule were realized by 3D Slicer software (www.slicer.org). Nodule segmentation was performed by two radiologists with more than 5 years of experience in chest CT imaging. We chose lung window levels of 1500 HU and -500 HU during nodule segmentation and drew the region of interest layer by layer. After nodule segmentation with 3D Slicer, radiomic features were calculated automatically by the software for each included case. These features are important for the prediction or diagnosis of disease and can be described as first-order features, shape and shape 2D features, texture features and wavelet features. Among them, textural features have been widely used in computer science and radiomics and can be used to represent the spatial distribution of areas with different intensities and can reflect tumour heterogeneity [15, 16]. Advanced algorithms can manage large feature quantities and select the most stable, reproducible ones to improve the efficacy of feature selection and the accuracy of classification. To guarantee the robustness of the above features, an intraclass correlation coefficient (ICC) cut-off was set for the test–retest analysis. An ICC greater than 0.80 was considered to indicate good interobserver consistency.

### Principal component analysis

We extracted 1130 radiomic features from each case. To avoid overfitting, principal component analysis (PCA) was used to select the optimal radiomic features and develop the prediction models by Python (version 3.6.5, www.python.org). The mechanism of PCA is to reduce the dimensionality of the data by identifying new variables, which are considered principal components (linear combinations of the original features). We selected principal components representing a cumulative contribution of 90% to construct the radiomics model.

### Histological evaluation

In this study, all of the included patients underwent resection of the lesions or biopsy. Samples were stained with haematoxylin and eosin (HE). The results of pathological lesions were re-evaluated by two experienced pathologists. If there was any disagreement, they discussed their findings with each other until a consensus was reached.

### Machine learning and model construction

The samples were randomly divided into a training cohort and validation cohort at a ratio of 7:3. We selected optimal radiomic features with the PCA method. After training with the logistic regression method, we constructed models of AP, VP and their combination (Model_AP_, Model_VP_, Model_Combination_). Then, ten-fold cross validation was applied in the validation cohort to test the performance of the models. Accuracy, precision and recall values were calculated. We compared the predictive performance of ten-fold cross validation by using the Wilcoxon signed-rank test. The area under the receiver operator curve (AUC) was used to evaluate the performance of the models.

### Statistical analysis

**Fig. 1:**
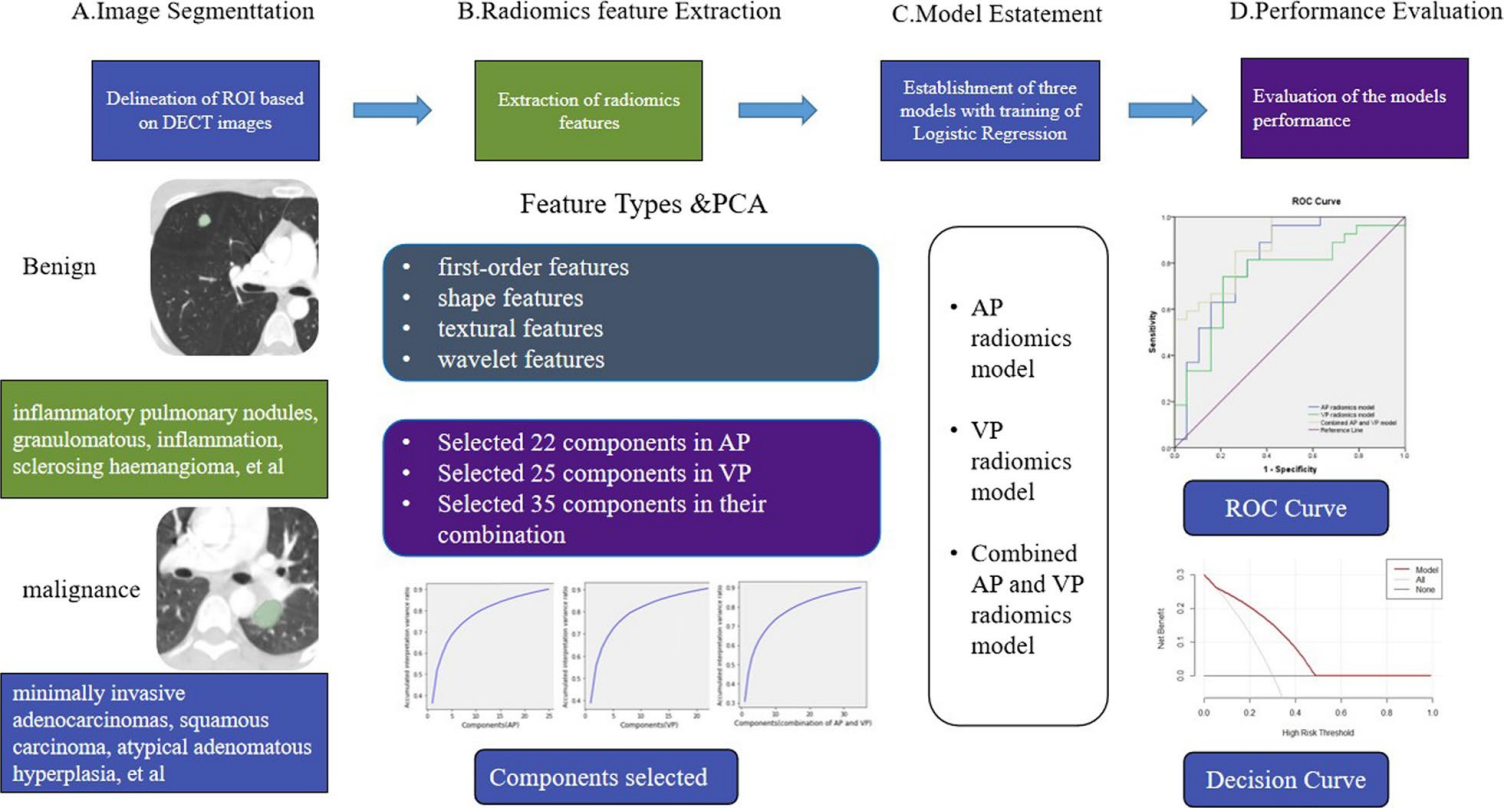
Flow chart of the radiomics analysis steps. Two radiologists manually segmented the region of interest (ROI) of pulmonary nodules. For model construction, the radiomic features were extracted, and principal component analysis was performed. The area under the curve (AUC) of the review operating characteristic was used to assess the diagnostic accuracy of the models. Decision curve analysis was used to assess the clinical utility of the models

The clinical data and features were analysed by SPSS 19.0 (SPSS, Chicago, IL, USA). The Wilcoxon rank sum test was used to compare normally and abnormally distributed continuous variables between the two groups. We calculated AUCs to assess the performance of the models in the primary and validation cohorts. The DeLong test was used for the comparison of AUCs in the validation set, and a difference was considered significant when *P* < 0.05 [17]. Decision curve analysis was used to assess the clinical utility of the model. The radiomics methods are included in Fig. 1.

## Results

### Patient features

In this study, all 153 patients had available pathological results. The histopathological examination revealed 53 cases of benign nodules (37 cases of chronic inflammatory pulmonary nodules, 5 cases of hamartoma, 2 cases of sclerosing haemangioma, 1 case of haemangioma, and 8 cases of granulomatous inflammation). The 100 malignant solitary pulmonary nodules included cases of 33 minimally invasive adenocarcinomas, 23 cases of squamous carcinoma, 19 cases of atypical adenomatous hyperplasia, 18 cases of adenocarcinoma in situ, and 7 cases of small-cell carcinoma. The included patients were divided into the training and validation cohorts (107 in the training cohort and 46 in the validation cohort). The baseline characteristics of the whole cohort are listed in Table 1. The average age of the patients was 47.97 years (range 23–68 years) in the whole cohort. A total of 70% of patients with malignant/benign (73/34) pulmonary nodules were assigned to the primary cohort, and 30% with malignant/benign (27/19) pulmonary nodules were assigned to the validation cohort. No significant difference in age was noted between the benign and malignant pulmonary nodule groups (*P* = 0.611). The size of the malignant nodules was significantly larger than that of the benign nodules (*P* = 0.006).

**Table 1:** The clinical characteristics of the patients

	Benign nodules (n = 53)	Malignant nodules (n = 100)	Whole set (n = 153)	*P* value
Age (years)	47.36 ± 11.125	48.30 ± 10.737	47.97 ± 10.846	0.611
Gender				
Male	32 (60.4%)	64 (64.0%)	96	
Female	21 (39.6%)	36 (36.0%)	57	
Nodule size (mm)	15.04 ± 5.248	17.58 ± 5.416	16.78 ± 5.477	0.006

### Radiomic features

**Fig. 2:**
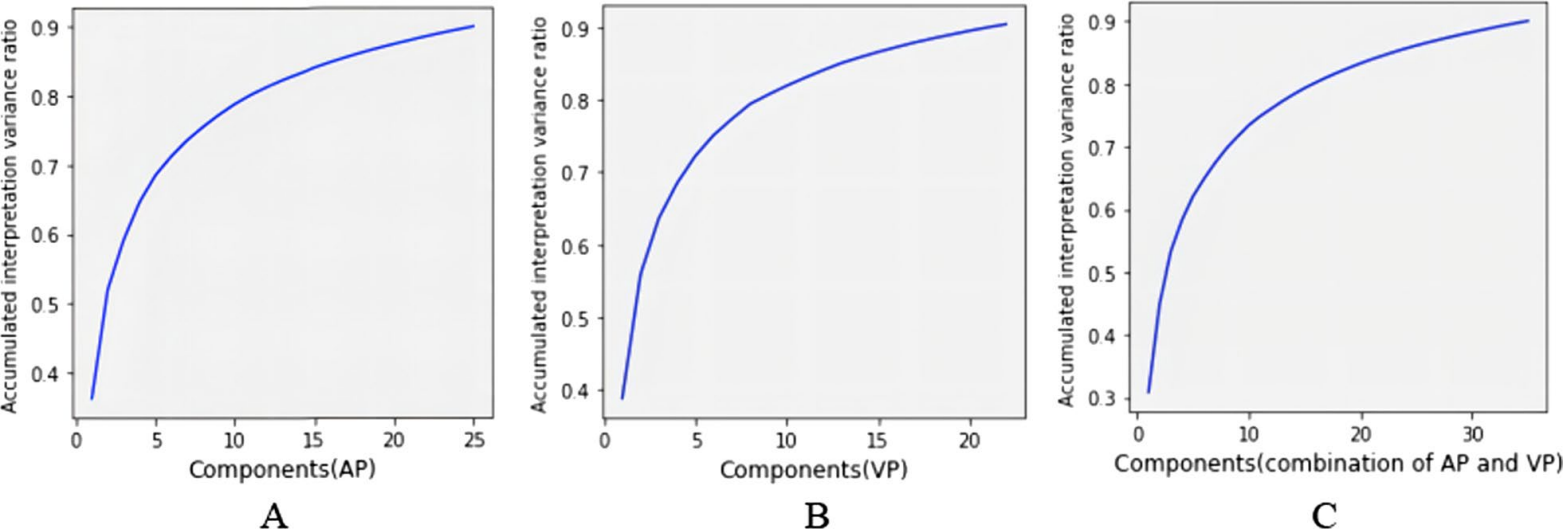
Line graph of the cumulative contribution rates of various principal components after selection from primary radiomic features. **A** Model_AP_, **B** Model_VP_, **C** Model_Combination_

In this work, the first-order features, shape and shape 2D features, texture features and wavelet features were extracted for each case. In total, 1130 radiomic features were extracted. Good interobserver consistency was achieved by the two independent experienced radiologists, with an interobserver coefficient greater than 0.80. For the radiomic features extracted from AP, the median ICC was 0.957 (interquartile range 0.904–0.976); 1081 of the 1130 features (95.7%) were robust. For the VP features, the median ICC was 0.914 (interquartile range 0.871–0.968); 1001 of the 1130 features (88.6%) were robust, with an ICC > 0.80, suggesting good consistency of feature extraction. The PCA method selected 22, 25, and 35 components in the arterial phase, venous phase and in the combination AP and VP, respectively, as shown in Fig. 2.

### Performance of the radiomics models

**Fig. 3:**
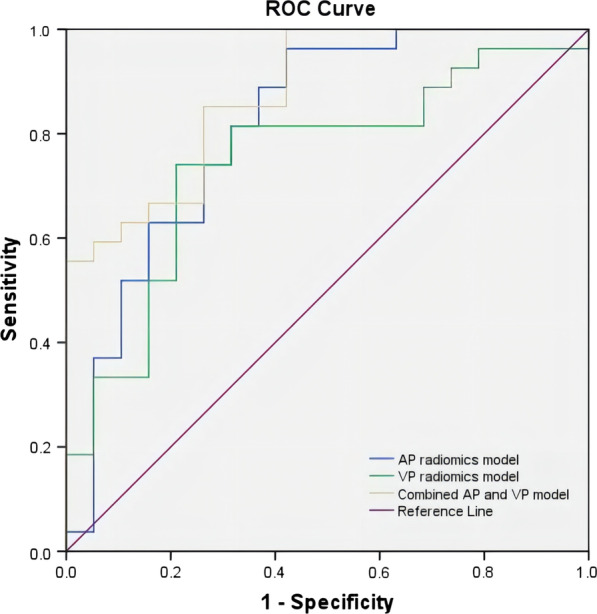
ROC curves of the AP radiomics model (blue line), VP radiomics model (red dotted line), and combined AP and VP radiomics model (purple line) in the discrimination of benign and malignant pulmonary nodules

**Fig. 4:**
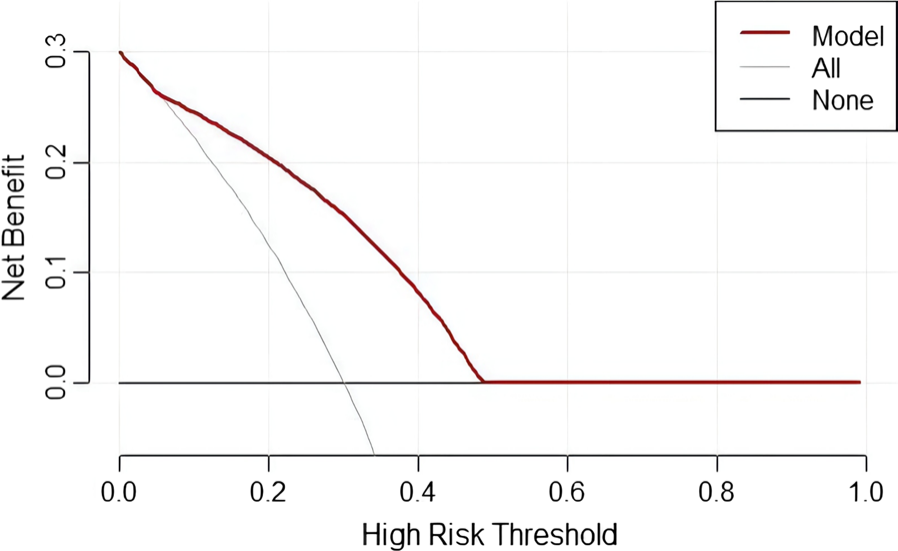
Decision curve analysis of the combined AP and VP model. The value of the y-axis represents the net benefit, and the x-axis represents the probability threshold. The results showed that when the threshold probability was within 0.06–0.50, the net benefit of the combined model was greater than that of the “all” and “none” schemes

After training with the logistic regression method, Model_AP_, Model_VP_ and Model_Combination_ showed good performance in the validation cohort, with AUCs of 0.8148 (95% CI 0.682–0.948), 0.7485 (0.602–0.895) and 0.8772 (0.780–0.974), respectively, and diagnostic accuracies of 0.8043, 0.6739 and 0.7826, respectively. Details are presented in Table 2. Receiver operator characteristic (ROC) curves are plotted in Fig. 3. To evaluate the difference in performance among the models in the validation cohort, the DeLong test was performed, and the results demonstrated that Model_Combination_ had better performance for discriminating benign from malignant pulmonary nodules (AUC = 0.8772, *P* < 0.05), with precision and recall of 75.76% and 92.59% in the validation cohort, respectively. Additionally, the Wilcoxon signed-rank test showed that the performance of Model_combination_ was significantly higher than the other models in ten-fold cross validation (*P* < 0.05). A total of 36 of 46 (78%) cases in the validation set were correctly predicted at the best cut-off value on the ROC curve. Decision curve analysis showed that when the threshold probability was within 0.06–0.50, the net benefit of Model_Combination_ was greater than that of the “all” and “none” schemes (Fig. 4).

**Table 2:** ROC analysis of the models in the validation set

	AUC (95% CI)	Accuracy	Precision	Recall	*P* value*
AP radiomics model	0.8148 (0.682–0.948)	0.8043	0.7647	0.9630	0.0396
VP radiomics model	0.7485 (0.602–0.895)	0.6739	0.6875	0.8148	0.0465
Combined AP and VP model	0.8772(0.780–0.974)	0.7826	0.7576	0.9259	/

*P* value*: DeLong test between the AP radiomics model and the combined model and between the VP radiomics model and the combined model

## Discussion

The advantage of radiomics in discriminating benign from malignant pulmonary nodules is the ability to identify quantities of characteristic features, which can help to depict the detailed imaging differences between them [18]. Some reports have described radiomics as an additional critical technique for comprehensively detecting tumour heterogeneity. It can also compensate for the shortcomings of traditional imaging diagnosis [19, 20]. Radiomics could be a valuable additional tool in cases where there is overlap in the results of morphological and perfusional analyses of malignant and benign features. The internal heterogeneity of malignant lesions is more commonly captured on contrast-enhanced scans, which can enhance the diagnostic accuracy for malignant nodules.

In recent years, many studies have been conducted to explore the use of conventional CT based on radiomic methods to distinguish benign and malignant pulmonary nodules [21–23], but few studies have focused on distinguishing pulmonary nodules using dual-energy CT based on radiomics. Xu et al. [24] developed a radiomic model based on conventional CT to differentiate benign from malignant lesions, different types of malignant nodules, and benign from non-invasive malignant nodules. The AUCs ranged from 0.84 to 0.89 in the test sets. Yao et al. [25] developed an LR model based on nonenhanced CT to predict malignant and benign nodules, with an AUC of 0.681. Meanwhile, the 70-keV monochromatic images have an advantage over the traditional CT in terms of beam-hardening artefact reduction and quantitative CT number accuracy. Furthermore, monochromatic images have less pattern noise [26, 27]. Thus, the quality of the obtained images is better, allowing for detailed imaging radiomic features to be extracted. In many studies exploring the use of DECT for identifying pulmonary nodules, most scholars selected 70-keV monochromatic images as the research object to achieve the best balance of image quality and radiation dose [3, 28]. The results of some recent studies have shown that DECT (70-keV monochromatic images) has better performance than conventional CT in discriminating benign from malignant pulmonary nodules and masses [3, 29].

Therefore, we constructed prediction models and trained them with the logistic regression method based on DECT monochromatic images (AP and VP) to further improve the accuracy of pulmonary nodule classification. The results of the present study demonstrated that these models could be used as an effective and non-invasive approach for discriminating benign from malignant pulmonary nodules before surgery. The best discrimination capability was found for the Model_combination_ rather than the Model_AP_ and Model_VP_, yielding an AUC of 0.8772 (95% CI 0.780–0.974). A recent study [30] showed that imaging at an energy level of 65 keV yielded the best diagnostic accuracy in identifying malignant solitary pulmonary nodules on AP and VP monochromatic images, with AUCs of 0.855 and 0.858, respectively. Compared with this study, the combination radiomics model (AUC of 0.8772) may show better performance at an energy level of 70 keV. In our study, the arterial phase and venous phase of dual-energy CT images were analysed based on the radiomic method used, with richer radiomics features being extracted and a larger amount of information being obtained from inside the lesion, making it highly valuable for model construction. The difference in intratumoral microvessel density between benign and malignant nodules, namely, the difference in capillary perfusion and permeability, will more prominent after contrast administration.

However, there are several limitations in this study that should be mentioned. First, this is a retrospective study with a small sample size, so selection bias is inevitable. Therefore, the generalizability and reliability of the results may be limited. In the future, large-sample prospective studies are needed to confirm these findings. Second, personal clinical experience impacted radiomic feature extraction, as the ROI was marked by manual segmentation. Third, there is higher radiation dose when performing AP and VP images for nodule detection compared with nonenhanced CT, However, contrast-enhanced CT provided additonal perfusion infromation, and homogenous enhancement may suggest the possibility of a benign vascular tumor. Finally, the clinical value of only the models constructed by principal component analysis was explored in distinguishing benign and malignant pulmonary nodules; clinical features were not included in this research. Therefore, we aim to further explore the performance of the radiomics model in combination with clinical features in the future.

## Conclusion

In conclusion, we developed a model based on monochromatic DECT images to identify solitary pulmonary nodules. The proposed model may serve as an effective tool for discriminating between benign and malignant pulmonary nodules. The combination of arterial phase and venous phase imaging could significantly improve the performance of the models.

## Data Availability

The datasets used and/or analysed during the current study are available from the corresponding author upon request. For ethical reasons, the raw data that we collected cannot be made publicly available. This study was approved by the review board of our institution (Hospital of Chengdu University of Traditional Chinese Medicine Research Ethics Committee). Access to the data was granted only to (1) members of the research team; (2) the Medical Ethics Committee members that approved this study; and (3) authorized personnel of the institution (Hospital of Chengdu University of Traditional Chinese Medicine Research Ethics Committee). However, requests for the anonymized data can be sent to prof. MG Xie at xmg6806@163.com.
